# No differences between remote and laboratory-based testing of cardiac interoceptive accuracy using the phase adjustment task

**DOI:** 10.1038/s41598-024-79125-4

**Published:** 2024-11-18

**Authors:** Ria Spooner, Jonathan M. Bird, Rhea Clemente, Nerea Irigoras Izagirre, Elisa Fernandez Fueyo, Dawn Watling, David Plans, Rebecca Brewer, Geoffrey Bird, Jennifer Murphy

**Affiliations:** 1 Department of Psychology, Royal Holloway, University of London, London, UK; 2 Department of Management, University of Exeter, Exeter, UK; 3 Department of Experimental Psychology, University of Oxford, Oxford, UK; 4 Department of Biological Sciences, Royal Holloway, University of London, London, UK; 5 Department of Anthropology, University College London, London, UK; 6 Centre for Research in Autism and Education, Institute of Education, University College, London, UK; 7 Department of Psychology, University of Surrey, Stag Hill, Guildford, UK

**Keywords:** Interoception, Interoceptive accuracy, Online testing, Remote testing, Photoplethysmography, Sensory processing, Human behaviour

## Abstract

In recent years, there has been an increased interest in remote testing methods for quantifying individual differences in interoception, the perception of the body’s internal state. Hampering the adoption of remote methods are concerns as to the quality of data obtained remotely. Using data from several studies, we sought to compare the performance of individuals who completed the Phase Adjustment Task—a new measure of cardiac interoceptive accuracy that can be administered via a smartphone application—supervised in a laboratory against those who completed the task remotely. Across a total sample of 205 individuals (119 remote and 86 laboratory), we observed no significant differences in task performance between the two groups. These results held when matching groups on demographic variables (e.g., age) and considering only individuals who had successfully completed a screener task. Overall, these data attest to the suitability of the Phase Adjustment Task for remote testing, providing an opportunity to collect larger and more diverse samples for future interoception research.

**Supplementary Information:**

The online version contains supplementary material available at 10.1038/s41598-024-79125-4.

## Introduction

With growing interest in interoception, the perception of the body’s internal state^1^, there has been an increased focus on measurement—particularly the measurement of interoceptive accuracy^2^. The measurement of individual differences in the ability to perceive bodily signals accurately presents a host of challenges well-covered in the literature (e.g^2–4^). However, comparatively less research attention is oriented toward the challenges associated with laboratory-based testing.

The measurement of interoceptive accuracy typically involves comparing an objectively recorded bodily signal (e.g., heartbeats) to participant reports^2^. Hence, to record bodily signals accurately, participants are often required to visit a laboratory to enable these physiological signals to be captured. This limits testing to participants who can travel to a laboratory, thereby reducing opportunities to test diverse samples of individuals. Additionally, this involves a large cost (both financial and in terms of participant and researcher time), impacting the opportunity to collect large datasets.

Acknowledging these challenges, interoception researchers have begun to explore the utility of remote testing methods such as webcams, smartwatches and smartphone applications (e.g^5–7^), which have recently been employed in experimental research (e.g^8,9^). The majority of these technologies have been used to implement tasks of cardiac interoceptive accuracy that may be contaminated by estimation strategies impacting validity (for discussion see^2^). Specifically, concerns have been raised that good performance may be achieved on certain tasks—for example the heartbeat counting task—via the use of informed guessing strategies, resulting in false positives (see^2^). An exception to this is the Phase Adjustment Task (PAT^6^). Throughout the PAT, participants are presented with tones that are triggered by their heartbeats, but are out of phase with those heartbeats. Participants are required to adjust a virtual dial until they believe the tone to be synchronous with their heartbeat. As the starting phase relationship between tones and heartbeats is random across trials, the consistency of participant responses is taken as a measure of interoceptive accuracy.

The PAT has been successfully implemented remotely in several samples (e.g^6,9^), using a camera-driven photoplethysmogram sensor that detects heartbeats when the participant places their finger over the smartphone camera and flash. The sensor detects changes in light intensity due to the pulse wave, and thus uses the same method as a conventional pulse oximeter. Nonetheless, concerns have been raised regarding the validity of data obtained using remote testing^2^. Central to these concerns is that remote testing may result in high measurement noise due to participants’ lack of compliance with task instructions, lack of control over the testing environment (e.g., increased distractions) and low reliability of heart rate capture. To date, however, no research has empirically examined whether these concerns are justified.

The aim of the present exploratory study was to compare the performance of individuals who completed the PAT under supervised laboratory conditions and individuals who completed the PAT unsupervised and remotely. To the best of our knowledge, this is the first study to systematically compare remote unsupervised and supervised laboratory-based data collection for a task of cardiac interoceptive accuracy. To achieve this aim, we made use of two available samples and explored the extent to which PAT scores and the proportion of individuals deemed interoceptive differed across the two samples, both with and without selecting participants according to their performance on a screening task.

## Methods

### Participants

All studies received ethical approval from the Royal Holloway Ethics Subcommittee following their reviewing procedures (see Supplement S3). In line with the Declaration of Helsinki, participants provided informed consent and were fully debriefed after task completion.

#### Laboratory sample

One hundred participants completed the PAT supervised in a laboratory as part of a larger pre-registered study involving multiple measures (Spooner et al., in prep; https://osf.io/j4dtr; for full recruitment and study details see Supplement S3 and S4). Participants were recruited via social media and Royal Holloway’s psychology participant pool and were only invited to participate if they were aged 18–65 years, with normal/corrected hearing and vision. Of these participants, 16 were excluded because too few valid trials were available for the screener task (< 17 valid trials based on engagement data. Note that 15–20 trials provides a reasonable trade-off between task duration and the expected consistency profile^6,9^. We set a threshold of 17 valid trials as this was the threshold pre-registered for the laboratory-based study see https://osf.io/j4dtr). For seven participants, the application had to be restarted from the beginning of the task due to technical issues (e.g., where the application crashed or could not detect the user’s heart beats). The majority of these participants had only completed practice trials before restarting, but one participant had completed a sizeable amount of the application before restarting and their first attempt was retained. One additional participant was excluded for having a screener score < 0.42 (see Materials and Procedure for task details), resulting in 83 laboratory participants with valid screener data. Note that a cut off value of 0.42 is taken from the probability function estimating the distribution of interoceptive and non-interoceptive participants from^6^ where the probability of being non-interoceptive is approximately 0 for a consistency score of 0.42. Given that the screener task of matching two tones is likely to be easier, a cut off of 0.42 for the screener is used to identify and exclude participants performing at a low level, indicative of a lack of task adherence or wider cognitive impairment.

Under the laboratory-based protocol, all participants (regardless of their screener scores) completed the PAT on the same day that they completed the screener task, separated by a small period of rest in which they completed a few questionnaires lasting approximately 20 min. Of participants passing the screener, 14 were excluded for completing too few trials. The application had to be restarted due to technical issues for four participants. However, very few trials had been completed prior to restarting and therefore the second attempt was retained for these participants. This resulted in a sample of 86 participants with valid PAT data.

Of the 86 participants with valid PAT data, 13 did not have valid screener data and were therefore excluded. This resulted in a final sample of 73 laboratory participants with valid data across the two (screener plus PAT) tasks (*M*_age_ = 22.90 years, *SD*_age_ = 7.0 years, 43 females).

##### Remote sample

Three hundred and seventy-seven participants took part in a remote study (based on accessing the application and completing a minimum of two trials) and were recruited via Testable. Although remote data were pooled from two projects (see Supplement S3 and S4 for details of each study), participants were required to own an Apple iPhone with a single camera across all studies due to software requirements for the application at the time the study was launched. Of these participants, 124 were excluded having completed too few trials for the screener or not having completed the screener task (< 17 valid trials). The application had to be restarted for 21 participants due to technical issues and their first attempts were retained in instances where 17 valid trials were available. However, we allowed later attempts if early attempts had fewer than the required number of trials (as with the laboratory study). Thirty-six participants were excluded for having a screener score < 0.42 (as per^6^), resulting in 217 remote participants with valid screener task data.

All participants were later invited to complete the PAT remotely, with participation being open for a period of approximately 2 years. One hundred and forty-three participants accessed the study (based on completing a minimum of 2 trials). Twenty-three participants were excluded for completing too few trials for the PAT (< 17 valid trials). The application had to be restarted for two participants due to technical issues and their first attempts were retained in instances where 17 valid trials were available. However, we allowed later attempts if early attempts had fewer than the required number of trials. One participant was excluded as they completed more than 20 trials due to a technical issue with the application. This resulted in 119 participants with valid PAT data.

Of the 119 participants with valid PAT data, 24 did not have valid screener data and were excluded. This resulted in a final sample of 96 participants with valid data across both the screener and the PAT (*M*_age_ = 30.98 years, *SD*_age_ = 9.2 years, 52 females). On average completion between the screener and PAT was 45 days, but this varied considerably across participants (*M* = 44.91 days, *SD* = 101.80 days, range 0–498 days).

#### Power analyses

As, to our knowledge, no study has yet compared remote unsupervised and supervised laboratory-based data collection for tasks of cardiac interoceptive accuracy, conducting a precise power analysis was not possible. We therefore powered for a medium effect size. However, as we made use of data from three studies, each with their own required sample size, our final sample size provided more power than planned for. The final sample size across the two datasets provides > 80% power to detect small-to-medium effect sizes (*d* = 0.35) for the primary analysis comparing PAT consistency scores across the two groups (i.e., laboratory vs. remote).

## Materials and procedure

After providing informed consent, each participant completed the screener task and PAT as described in^6^, implemented using a purpose-built smartphone application. In both tasks, participants were required to lay their hand flat on the table with their finger over the smartphone camera and flash and heartbeats were recorded via photoplethysmography. After a 2-min baseline recording period to capture heart rate, participants were presented with task instructions relevant to the task they were completing (see Supplementary Materials S1 and S2 for full instructions). In the PAT, participants are presented with a series of tones that are triggered by their heartbeat, but out of phase with their heartbeat. They are required to adjust a virtual dial to advance or delay the tones in time until they perceive them to be synchronous. After confirming their response, participants are prompted to rate their confidence in having successfully completed the trial on a 10-point scale (0 = “Not at all confident”, 9 = “Extremely confident”) and are then automatically advanced to the next trial. Every 5 trials, participants are also presented with a body map on which they are asked to indicate the location from which they felt their heartbeat on the previous trial. The screener task was identical to the PAT, except that participants were presented with two tones on each trial, one triggered by their heartbeat and the other triggered by the heartbeat but out of phase with the heartbeat and were required to adjust the dial until they perceived the two tones to be synchronous (see^6^; Study 2). Note that tones were triggered by heartbeats in both tasks. This differs from the original PAT study^6^ where an algorithm was used to predict the occurrence of the next heartbeat from the preceding 3 s recording. This change from using predicted to actual heartbeats was implemented due to the possibility that the accuracy of the algorithm’s predictions may vary slightly across participants as a function of heart rate variability. Triggering tones from heartbeats removed the possibility of differences in the accuracy of predictions. For both tasks, participants completed 2 practice trials, followed by 20 task trials.

Testing procedures varied slightly across the participant groups. Regarding the laboratory study, participants completed the screener task and the PAT on the same day, separated by a small period of rest. They were supervised by either RS, RC or NII and completed the tasks on Apple iPhones provided by the researchers. Participants were recruited via Testable for the remote study. Data were pooled from two projects for the purpose of this study to make use of all available data collected remotely for studies implemented by JM (Sample 2) and EFF (Sample 3). For all projects, participants were initially screened using either Gorilla or Qualtrics to ensure their smartphone met the application requirements. A small number of participants completed questionnaires and a behavioural task on Gorilla prior to completing the task (Sample 3). After determining eligibility, participants were presented with the instructions for downloading the applications. The majority of participants were then only invited to complete the PAT application at a later time if they had completed the screener application to a sufficient standard (as described above), though a small minority of remote participants did not complete the screener and were only included in an additional analysis (described below) and a further small minority completed the PAT on the same day as the screener separated by approximately 30 min of tasks and questionnaires.

### Analysis strategy

Data were initially stored in JSON format consisting of multiple nested lists containing key-value pairs. We imported the JSON data into RStudio (v2023.12.1) and sought to unnest the data using the tidyr package, rendering the data suitable for further analyses in tidy format (i.e., each row corresponding to a PAT trial). Following extraction of the beat-to-beat (RR) intervals from the 2 min baseline heart rate data, we used the RHRV R package to calculate several time domain heart rate variability metrics (i.e., RMSSD, pNN50, SDNN). Engagement metrics were also computed on a trial-by-trial basis (i.e., number of unique dial positions, time taken to completion).

After removing each participant’s practice trials (*n* = 2), we applied a range of quality checks on the remaining data. We removed trials that contained ≤ 4 heart rate values, as this indicated a lack of engagement with the task. Similarly, we removed trials that contained 0 delays, as this meant that the dial position had remained unchanged from its default position. As pre-registered, we excluded participants with < 17 valid trials out of the 20 possible trials. To ensure all participants had equal trial numbers, we selected each participant’s first 17 trials (while discarding additional trials) to allow for the computation of aggregate engagement metrics (e.g., mean time spent on trials; mean number of dial turns; number of valid trials) and the comparison of consistency scores.

Consistency scores refer to the consistency of the selected delays across PAT trials and are used to help determine whether an individual is interoceptive or non-interoceptive. Briefly, consistency scores are computed from measures of angular similarity representing the phase relationship between heartbeats and tones on a trial-by-trial basis. If angles are close to one another, the corresponding consistency score will be close to 1. On the other hand, if angles are randomly positioned, the corresponding consistency score will be close to 0 (see^6^ for additional details).

We used the AdaptGauss package to apply a gaussian mixture model to the consistency scores to classify participants as either interoceptive or non-interoceptive. The mixture model returned two distributions, one for interoceptive and one for non-interoceptive participants by means of an expectation-maximisation algorithm^6^. We calculated *z*-scores for each participant for interoceptive and non-interoceptive distributions separately. Thereafter, the *z*-scores were used to calculate the probability of each participant belonging to the interoceptive and non-interoceptive distributions, as in^6^. Bayes Factors (BF) were calculated across the entire sample (i.e., laboratory and remote) as the ratio of the probability of belonging to one of the two distributions over the probability of belonging to the other distribution. Thresholds were used on the BFs, which meant that participants could be classified as interoceptive, non-interoceptive, or unclassified (i.e., where there is insufficient evidence for a classification). BFs > 3 provided moderate evidence that a participant was interoceptive or non-interoceptive, BFs > 10 provided strong evidence, and BFs > 30 provided very strong evidence^6,9^.

Data were then imported into SPSS for formal analyses and can be accessed at (https://osf.io/uf3ap/). Formal analyses compared mean consistency scores between laboratory and remote participants using independent samples *t*-tests and classification scores using chi-square analyses. Normality assumptions were checked using visual inspection and where deviations from normality were observed (for engagement metrics and heart rate metrics), non-parametric equivalents were used. One outlier was present for consistency scores, but was retained as excluding this participant did not alter the pattern of significance.

## Results

For demographics, engagement metrics, and heart rate data for both samples, please see Tables 1 and 2.

**Table 1:** Demographic variables, engagement metrics and heart rate data for both screened samples before and after matching.

	Screened sample unmatched for age				Screened sample matched for age				
	Laboratory	Remote	Z	*p*	Laboratory		Remote	Z^4^	*p*
	M (SD)	M (SD)			M (SD)		M (SD)		
Mean time per trial (s)	21.64 (10.93)	24.06 (13.02)	0.985	0.324	23.08 (11.84)		24.74 (12.89)	0.494	0.621
Mean engagement (dial turns) per trial	27.65 (15.39)	27.97 (14.96)	0.225	0.822	29.42 (16.97)		28.63 (14.46)	0.004	0.997
Number of valid trials	19.16 (0.972)	19.08 (1.073)	0.333	0.739	19.06 (1.05)		19.04 (1.13)	0.046	0.963
Resting heart rate (bpm)	80.73 (10.57)	74.17 (10.35)	4.040	**< 0.001**	80.12 (12.16)		74.40 (10.23)	2.208	**0.027**
Heart rate variability (SDNN)	179.90 (79.63)	152.55 (95.36)	2.523	**0.012**	187.25 (72.24)		163.49 (96.71)	1.703	0.089
Heart rate variability (RMSSD)	148.30 (63.51)	123.56 (70.39)	2.723	**0.006**	156.84 (57.35)		126.44 (61.88)	2.713	**0.007**
Heart rate variability (pNN50)	52.01 (18.55)	47.80 (20.07)	1.360	0.174	53.73 (18.49)		51.42 (19.43)	0.635	0.525
Age	22.90 (6.99)	30.98 (9.18)	6.712	**< 0.001**	24.39 (5.10)		23.54 (2.79)	0.225	0.822

	N Males (N Females)	N Males (N Females)	X^2^	*p*	N Males (N Females)		N Males (N Females)	X^2^	*p*
Sex	30 (43)	44 (52)	0.378	0.539	23 (26)		22 (26)	0.012	0.913

As the majority of data were not normally distributed, non-parametric tests (Mann–Whitney U) were used for all comparisons.

**Table 2:** Demographic variables, engagement metrics and heart rate data for both unscreened samples before and after matching.

	Unscreened sample unmatched for age				Unscreened sample matched for age			
	Laboratory	Remote	Z	*p*	Laboratory	Remote	Z^6^	*p*
	M (SD)	M (SD)			M (SD)	M (SD)		
Mean time per trial (sec)	21.24 (10.64)	23.87 (13.15)	1.175	0.240	22.07 (11.53)	24.00 (12.10)	0.896	0.370
Mean engagement (dial turns) per trial	27.06 (15.10)	28.39 (15.98)	0.608	0.543	28.10 (16.61)	28.24 (13.88)	0.536	0.592
Number of valid trials	19.16 (1.00)	19.00 (1.09)	1.054	0.292	19.16 (1.07)	19.00 (1.11)	0.834	0.404
Resting heart rate (bpm)	80.18 (11.20)	75.64 (10.51)	3.176	**0.001**	79.72 (12.67)	75.45 (10.31)	1.997	**0.046**
Heart rate variability (SDNN)	178.10 (78.05)	154.43 (98.04)	2.505	**0.012**	180.53 (71.11)	160.13 (96.13)	1.749	0.080
Heart rate variability (RMSSD)	146.89 (61.09)	119.14 (67.79)	3.428	**< 0.001**	153.81 (57.19)	122.38 (61.32)	3.020	**0.003**
Heart rate variability (pNN50)	52.82 (17.75)	45.41 (20.73)	2.521	**0.012**	54.28 (17.69)	49.93 (20.04)	1.104	0.270
Age	23.09 (7.71)	31.01 (9.36)	7.304	**< 0.001**	24.78 (6.74)	23.73 (2.75)	0.203	0.839

	N Males (N Females)	N Males (N Females)	*X* ^**2**^	*P*	N Males (N Females)	N Males (N Females)	*X* ^**2**^	*p*
Sex	33 (53)	53 (63)	1.082	0.298	26 (32)	25 (35)	0.120	0.729

As the majority of data were not normally distributed, non-parametric tests (Mann–Whitney U) were used for all comparisons. Demographic data was missing for 3 participants from the remote study.

Examination of the consistency scores revealed identical means and standard deviations across the two samples (*M* = 0.37, *SD* = 0.17), rendering statistical analysis comparing consistency scores across the two samples unnecessary. When considering the classification of participants as interoceptive, non-interoceptive, and unclassified, results did not significantly vary across the two groups (see Table 3a).

**Fig. 1:**
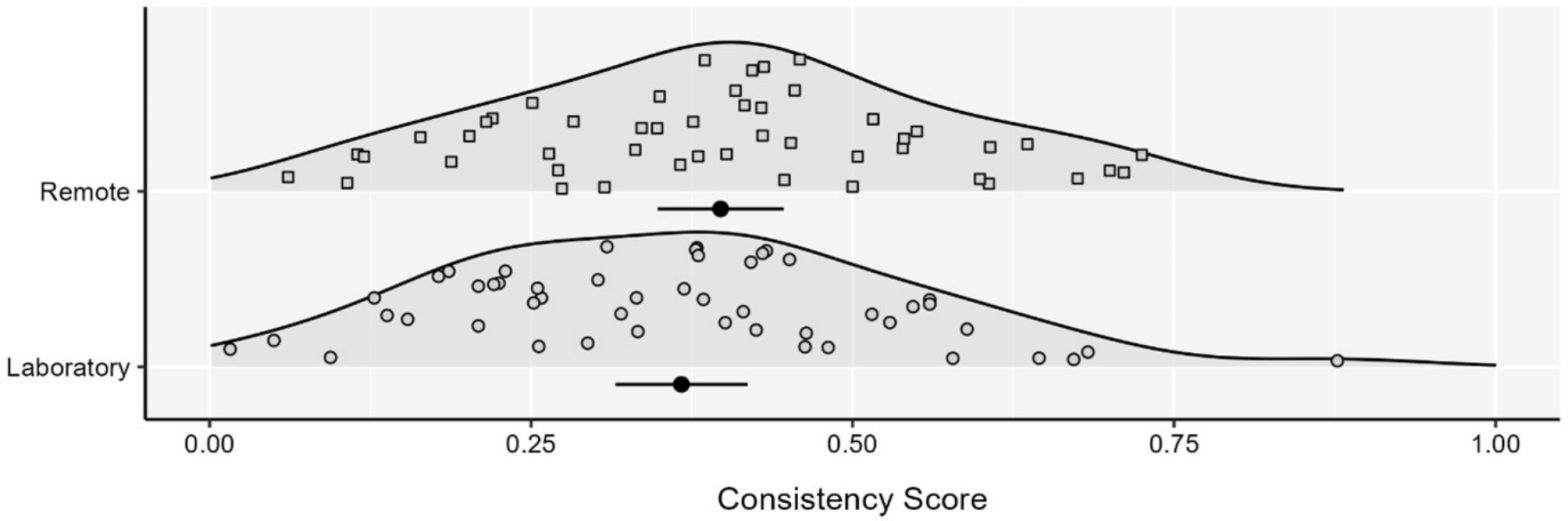
Consistency scores for screened participants between data collection methods. Each density is accompanied by the mean and 95% CI. Figure depicts scores after groups were matched for age. Laboratory *N* = 49. Remote *N* = 48.

As laboratory and remote samples differed with respect to age (see Table 1), we took the following approach to match samples for age. As the majority of participants for the laboratory study were aged between 18 and 21 years (56.2% compared to 11.5% for the remote study), to match groups on average we excluded participants aged 30 years and over from the remote cohort and excluded participants under 19 years from the laboratory cohort as well as one participant from the laboratory cohort who was an outlier in terms of age (aged 63 years) (see Table 1). After matching groups, there was still no significant difference in consistency scores between laboratory (*M* = 0.37, *SD* = 0.18) vs. remote (*M* = 0.40, *SD* = 0.17) participants (*t*(95) = 0.863, *p* = .39; see Fig. 1). When considering the classification of participants as interoceptive, non-interoceptive, and unclassified, results again did not vary across groups after age matching (see Table 3b). In this age matched sample, we observed no differences in engagement data (total time spent on trials; mean time spent on trials; mean number of dial turns; number of valid trials; see Table 1), but there were differences in resting heart rate and heart rate variability RMSSD (both *p* < .05; Table 1). As a final control analysis, we regressed out heart rate and heart rate variability (RMSSD) from consistency scores. Again, we found a non-significant difference between the performance of laboratory vs. remote participants (*t*(95) = 0.748, *p* = .456).

**Table 3:** Classification of participant scores for screened participants.

Dataset	Laboratory (*N* = 73)			Remote (*N* = 96)			X^2^	*p*
(a)	Non-interoceptive	Unclassified	Interoceptive	Non-interoceptive	Unclassified	Interoceptive		
BF3	26	17	30	36	21	39	0.079	0.961
BF10	12	40	21	15	58	23	0.612	0.737
BF30	0	63	10	0	80	16	0.281	0.596

	Laboratory (*N* = 49)			Remote (*N* = 48)				
(b)	Non-interoceptive	Unclassified	Interoceptive	Non-interoceptive	Unclassified	Interoceptive	X^2^	*p*
BF3	18	11	20	14	10	24	0.901	0.637
BF10	8	29	12	6	28	14	0.447	0.800
BF30	0	41	8	0	40	8	0.002	0.964

Panel a presents data in the full samples. Panel b presents data in the samples group matched for demographics.

We repeated the aforementioned analysis on the full sample data, including all individuals with valid PAT data, regardless of their screener performance. Data were available from 119 participants for the remote study (M_age_ = 30.10 years, SD_age_ = 9.36 years, 63 females; 3 missing demographic information) and 86 participants for the laboratory study (M_age_ = 23.09 years, SD_age_ = 7.71 years, 64 females). Results revealed a non-significant difference in consistency scores when comparing laboratory (*M* = 0.36, *SD* = 0.17) and remote (*M* = 0.37, *SD* = 0.17) participants (*t*(203) = 0.510, *p* = .61). When considering the classification of participants as interoceptive, non-interoceptive, and unclassified, we did not find any significant differences across groups (Table 4a).

**Fig. 2:**
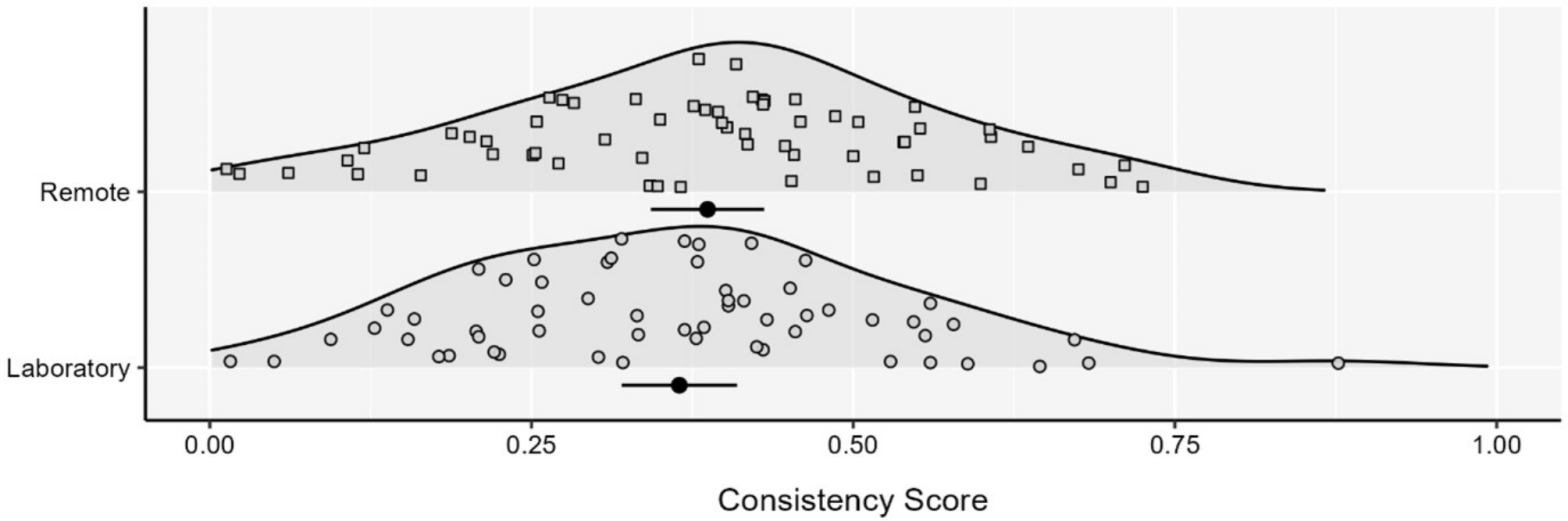
Consistency scores for unscreened participants between data collection methods. Each density is accompanied by the mean and 95% CI. Figure depicts scores after groups were matched for age. Laboratory *N* = 58. Remote *N* = 60.

As before, age differed between groups (Table 2). To match age, we used the same process described above and also excluded three participants for whom demographic data were unavailable for the laboratory study; see Table 2). After matching age at the group level, there were again no significant differences in consistency scores comparing laboratory (*M* = 0.37, *SD* = 0.17) and remote (*M* = 0.39, *SD* = 0.17) participants in the age matched sample (*t*(116) = 0.699, *p* = .486; see Fig. 2). When considering the classification of participants as interoceptive, non-interoceptive, and unclassified, there was again no difference across groups after age matching (Table 4b). As before, in the age matched sample there was no significant difference between groups in engagement data (Table 2), but differences in heart rate and heart rate variability RMSSD were observed (*p* < .05; Table 2). After controlling for these differences, there remained no significant difference in performance between laboratory and remote participants (*t*(116) = 0.753, *p* = .453).

**Table 4:** Classification of participant scores including unscreened participants.

Dataset	Laboratory (*N* = 86)			Remote (*N* = 119)			X^2^	*p*
(a)	Non-interoceptive	Unclassified	Interoceptive	Non-interoceptive	Unclassified	Interoceptive		
BF3	31	23	32	43	28	48	0.333	0.847
BF10	15	49	22	17	73	29	0.508	0.776
BF30	0	76	10	0	100	19	0.774	0.379

	Laboratory (*N* = 58)			Remote (*N* = 60)			X^2^	*p*
(b)	Non-interoceptive	Unclassified	Interoceptive	Non-interoceptive	Unclassified	Interoceptive		
BF3	20	16	22	18	13	29	1.343	0.511
BF10	9	36	13	8	35	17	0.573	0.751
BF30	0	50	8	0	52	8	0.005	0.942

Panel a presents data in the full samples. Panel b presents data in the samples group matched for demographics.

## Discussion

The aim of the present study was to compare the performance of individuals completing the PAT supervised in a laboratory setting and unsupervised remotely. To our knowledge, this is the first study to systematically compare remote unsupervised and supervised laboratory-based data collection for a task of cardiac interoceptive accuracy. Across both continuous and categorical classification scores, matched and unmatched samples, and screened vs. unscreened participants, we found no evidence of differences across the laboratory and remote samples. Overall, these results provide reassurance that the PAT can be administered remotely in an unsupervised fashion, and that data collected remotely is comparable to those collected in a supervised laboratory environment. It has been suggested that remote unsupervised testing may create additional noise^2^. However, we found no evidence that remote testing impacted PAT scores, even when unsupervised.

The data presented herein attest that the PAT can be administered remotely where required, but it should be acknowledged that there are trade-offs for laboratory vs. remote testing. Remote testing reduces demands on researcher time and may make studies more accessible to participants who cannot travel to a laboratory (for example, clinical groups^9^), increasing the diversity and size of samples, but remote testing also creates some disadvantages. Indeed, despite these advantages, it is clear from the completion rates across the two samples that far more participants who accessed the application remotely did not complete the application compared to those who completed the study in a laboratory and more participants experienced technical issues. Similar to issues with selection bias that may be present for laboratory studies, it may be that individuals who successfully complete the PAT remotely differ from those who cannot. However, there is no reason to expect that this would change the number of interoceptive individuals present in the sample, as to our knowledge there are no known variables that covary with potential selection variables and interoceptive accuracy. Nevertheless, it is worth acknowledging that remote testing may be inappropriate for researchers recruiting rare samples, as the amount of data loss may be prohibitive, as well as samples with less digital literacy. Future work to improve the participant on-boarding experience may help to mitigate these issues. In addition, participants could be supervised while completing the PAT remotely, through videoconference software where available.

It is also noteworthy that participants completed the PAT application via a smartphone for both the laboratory and remote studies. This ensured a similar participant experience (i.e., the requirement for a specific hand position for heart rate capture), but it should be acknowledged that these data cannot speak to the reliability of data capture using photoplethysmography when implemented via smartphone^2^. The reliability of such methods for heart rate capture is well-established^10,11^, and these methods have been used in various studies of interoception^6,9^. Nonetheless, it is presently unclear whether PAT performance varies when comparing the gold-standard ECG assessment against photoplethysmography, though there is no reason to expect that it would.

It is also worth acknowledging that, at present, the application is programmed for use with iOS devices, making it inaccessible to Android users for remote testing. However, a cross-platform version is currently in preparation (see https://github.com/davidplans/veris), which will further support the recruitment of remote samples. These data provide reassurance that the recruitment of samples via remote methods does not change the proportion of interoceptive individuals, making the development of a cross-platform version worthwhile. Indeed, the development of remote testing methods may be particularly advantageous for studies involving clinical groups, and others who may find it difficult to visit the laboratory^9^.

More broadly, despite challenges measuring cardiac interoceptive accuracy (and interoception more broadly across domains and dimensions; see^2,3^), these data speak to the utility of the PAT as a measure of cardiac interoceptive accuracy, and further underscore the suitability of the measure for both laboratory and remote testing. Indeed, whilst the PAT suffers from some of the same issues as all tasks of interoceptive accuracy (for example, difficulties separating signal strength from perception), the PAT overcomes several issues present in existing tasks, such as the use of estimation strategies. The task also controls for individual differences in the point in time at which individuals perceive an external stimulus to be synchronous with their heartbeat (participants must be consistent across trials, not perceive their heartbeat at a point in the cardiac cycle predetermined by researchers; for discussion see^2^) and the task is not influenced by physiological or psychological confounds). These features of the task mean that it has been described as one of the most promising new measures for the assessment of cardiac interoceptive accuracy^2^. These data further underscore its utility, showing no evidence of increased noise when participants are tested remotely and unsupervised.

In summary, we found no evidence of differences in PAT performance when comparing participants who completed the task supervised in a laboratory setting or unsupervised remotely. Remote testing may not be appropriate for all studies and more work is required to improve participant on-boarding. Nonetheless, these data speak to the suitability of the PAT for remote testing where required, opening up the possibility of diversifying samples and increasing sample sizes in future interoception research, as well as facilitating studies in clinical groups who may find it difficult to visit the laboratory.

## Electronic supplementary material

Below is the link to the electronic supplementary material.

Supplementary Material 1

## Data Availability

Data can be accessed at https://osf.io/uf3ap/.
