# Supplementary material for: No differences between remote and laboratory-based testing of cardiac interoceptive accuracy using the phase adjustment task
Source: Sci Rep. 2024 Nov 18;14:28524. doi: 10.1038/s41598-024-79125-4 (PMC11574115; doi:10.1038/s41598-024-79125-4)
Supplement: Supplementary file 1 — Supplementary Material 1 [file 41598_2024_79125_MOESM1_ESM.docx]

**Supplementary Material**

**S1. Transcription of screener instructions**

Please note that participants were free to move back and forth between the different instruction screens.

Screen 1: “How well can you match two sounds using a dial? Lets find out!”

Screen 2: “Find a quiet place where you can sit comfortably upright for around 10 minutes. We’ll be recording your heartbeats, so you therefore need to keep your hand still and in the correct position. Also, make sure you turn your phone’s volume up and don’t use earphones (plugged or bluetooth).”

Screen 3: “Getting ready to check your heartbeat. We will shortly turn on the LED Flash and camera on this phone, and will use it to take your heart rate. Please place your index finger across both camera and flash.”

Screen 4: [HEART RATE BASELINE READING]

Screen 5: “Your task is simple. You will hear two beeps. At the start there will be a delay between the beeps- you will hear one, and then after a short while you will hear the other. Your job is to try and get them in sync; we want you to get them to occur at exactly the same time. You do this by turning the dial on the screen with your finger. If you start with your finger at ’12 o’clock’ you can rotate the dial, keeping your finger on the screen at all times. At one point on the dial the tones will be in sync- they will happen at the same time. Sometimes, at random, the two beeps might appear to be in sync at the start. If this occurs, it’s best if you try turning the dial a little bit one way and then another, to check whether two beeps are really occurring at exactly the same time.”

Screen 6: You will now get a chance to do two practice trials.

**S2. Transcription of Phase Adjustment Tasks instructions**

Please note that participants were free to move back and forth between the different instruction screens.

Screen 1: “How well can you match a sound with your heartbeat? Let’s find out!”

Screen 2: “First, find a quiet place where you can sit comfortably upright with your earphones on for around 10 minutes. We will begin by capturing a couple of minutes of your heart rate, to get a sense for how your heart does its thing.”

Screen 3: “Getting ready to check your heartbeat. We will shortly turn on the LED Flash and camera on this phone, and will use it to take your heart rate. Please place your index finger across both camera and flash.”

Screen 4: [HEART RATE BASELINE READING]

Screen 5: “Ok, thanks! In the main task, you will be asked to place your finger on the phone camera (on the back) so that the app can read your heartbeat. Once your finger is in position, you will hear a series of sounds. Each sound actually represents one of your own heartbeats!”

Screen 6: “It might seem like there is a delay between the sounds and the heartbeats you feel. Play the video below to hear an example! [VIDEO 1; see Plans et al., 2021]”

Screen 7: “In order to rectify the delay, you will be asked to move a dial until the sounds are in sync with your heartbeats. Play the video below to hear an example! [VIDEO 2; see Plans et al., 2021].”

Screen 8: “Your objective is to find the point on the dial where the heartbeat and sound is in sync, by turning the dial left or right.”

Screen 9: “Want to know how this might look like? Press “continue” to watch a short tutorial.”

Screen 10: [VIDEO 3; see Plans et al., 2021]

Screen 11: “After you have matched the sound with your heartbeat, you will be asked how sure you are about the answer you gave. Press “confirm” then “continue” to start the following trial. In this task, there will be 20 trials in total. [EXAMPLE OF THE CONFIDENCE SCALE; see Plans et al., 2021]”

Screen 12: “You can feel your heartbeat in different places in your body, such as your chest or yours fingers. You will be asked to indicate where you felt your heartbeat on a body map (like the one below) once every 5 trials. You can choose any of the highlighted body parts or you can select “nowhere” if you haven’t felt your heartbeat in any particular place. [EXAMPLE OF THE BODY MAP; see Plans et al., 2021]”

Screen 13: “For the duration of this task, please do not actively try to feel your pulse with your hand; we are only interested in what you feel! You might feel your heartbeat in various bodily locations. Just make sure you pick one and stick to using the one during the task. When you are ready to start, please sit comfortably upright with your earphones on and press “continue”.”

Screen 14: “You will now get a chance to do two practice trials. Focus on feeling your heartbeat and try to match the sounds to your own heartbeat."

**S3. Sample information**

|  | Sample 1 | Sample 2 Sample 3 | | |
| --- | --- | --- | --- | --- |
| Data collection | Laboratory | Remote | Remote | |
| Ethical approval ID | 2186 | 3056 | | 3090 |
| Inclusion and exclusion criteria | Aged 18–65 years. Normal/corrected hearing/vision. | Aged 18–60 years. Access to an Apple iPhone meeting eligibility requirements. Normal/corrected hearing/vision. | | Aged 18–60 years. Access to an Apple iPhone meeting eligibility requirements. Normal/corrected hearing/vision. |
| Testing procedure | PAT and Screener completed on the same day, separated by self-reported interoception and mental health questionnaires. | Screener completed and eligible participants contacted to complete the PAT. | | Screener completed and eligible participants invited to complete the PAT. Participants completed questionnaires of self-reported interoception and empathy and an empathy task before the PAT. |

*Note.* PAT = Phase Adjustment Task.

**S4. Flow Diagram depicting sample recruitment and selection**

**
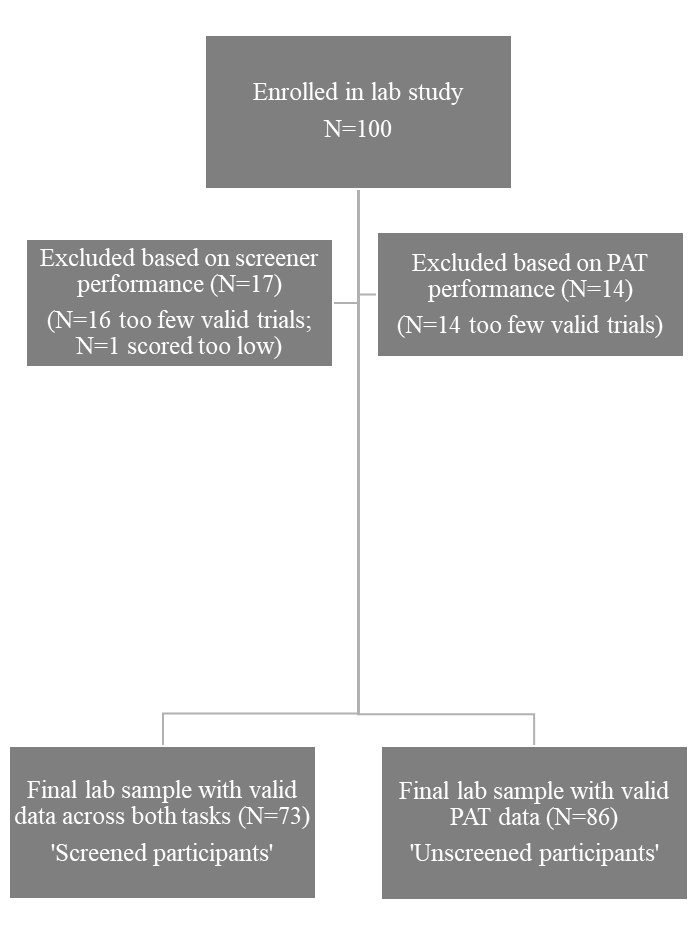

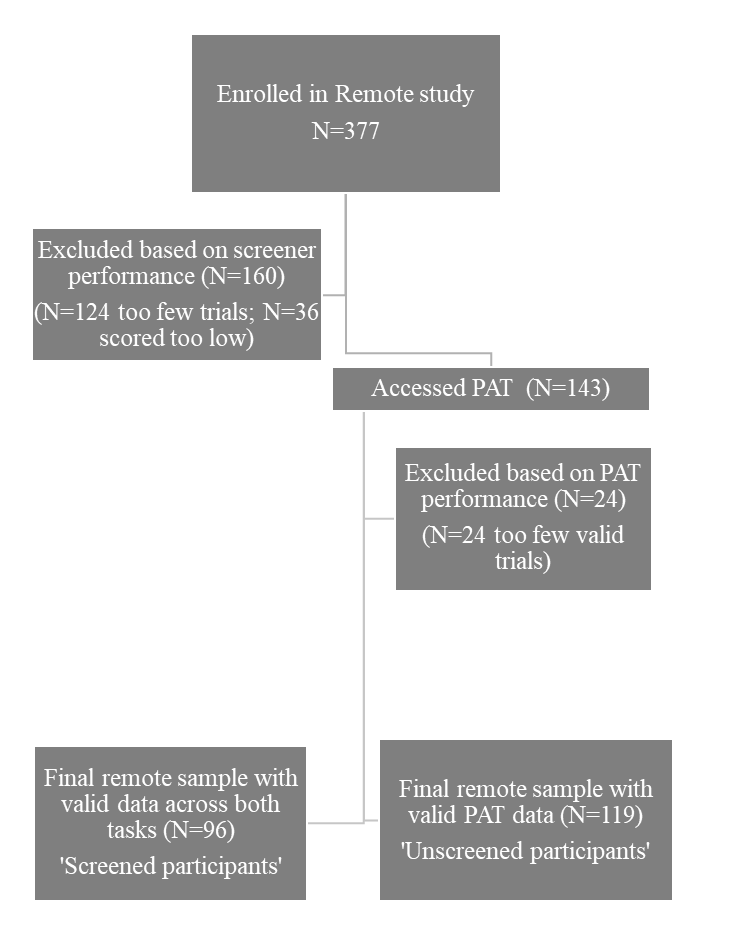
**
